# Who Gets the Last Bed? A Discrete-Choice Experiment Examining General Population Preferences for Intensive Care Bed Prioritization in a Pandemic

**DOI:** 10.1177/0272989X21996615

**Published:** 2021-03-04

**Authors:** Amelia E. Street, Deborah J. Street, Gordon M. Flynn

**Affiliations:** Intensive Care Unit, Prince of Wales Hospital, Randwick, New South Wales, Australia; Centre for Health Economics Research and Evaluation (CHERE), University of Technology Sydney, Haymarket, New South Wales, Australia; Intensive Care Unit, Prince of Wales Hospital

**Keywords:** clinical practice guidelines, discrete choice experiment, intensive care, resource allocation

## Abstract

**Objective:**

To explore the key patient attributes important to members of the Australian general population when prioritizing patients for the final intensive care unit (ICU) bed in a pandemic over-capacity scenario.

**Methods:**

A discrete-choice experiment administered online asked respondents (*N* = 306) to imagine the COVID-19 caseload had surged and that they were lay members of a panel tasked to allocate the final ICU bed. They had to decide which patient was more deserving for each of 14 patient pairs. Patients were characterized by 5 attributes: age, occupation, caregiver status, health prior to being infected, and prognosis. Respondents were randomly allocated to one of 7 sets of 14 pairs. Multinomial, mixed logit, and latent class models were used to model the observed choice behavior.

**Results:**

A latent class model with 3 classes was found to be the most informative. Two classes valued active decision making and were slightly more likely to choose patients with caregiving responsibilities over those without. One of these classes valued prognosis most strongly, with a decreasing probability of bed allocation for those 65 y and older. The other valued both prognosis and age highly, with decreasing probability of bed allocation for those 45 y and older and a slight preference in favor of frontline health care workers. The third class preferred more random decision-making strategies.

**Conclusions:**

For two-thirds of those sampled, prognosis, age, and caregiving responsibilities were the important features when making allocation decisions, although the emphasis varies. The remainder appeared to choose randomly.

## Introduction

The COVID-19 pandemic is on its way to becoming the greatest health crisis of this century. It has overwhelmed intensive care units (ICUs) and hospitals in many countries, from the outset in Wuhan, to Italy, and then on to affect Spain, France, the United Kingdom, Brazil, and the United States of America, to name just a few.^1,2^ At the time of writing, Australia has faced a second wave and is trying to manage outbreaks, while to date, New Zealand has avoided this confronting state of affairs. Both locally and internationally, guidelines have been prepared to help guide decision making when ICU demand exceeds capacity.^3–5^ These guidelines reflect the fact that when health care systems are overwhelmed, an individual’s health care needs cannot be considered in isolation, but rather resource allocation must balance each individual’s needs with delivering the best outcome for the broader community.^6,7^

This is not the first time that rationing has been considered in modern health care systems. Organ donation services have grappled with fairly rationing access to organs for more than 50 y, and the topic remains contentious. Discourse in this area has highlighted the need to avoid discrimination based on irrelevant grounds, including avoiding value judgments about a patient’s profession or social standing.^8^ Emanuel et al.^7^ discussed different approaches in the context of this pandemic, such as aiming to maximize the life-years saved, prioritizing those who are likely to continue to assist in the pandemic care or recovery, such as health care workers, or rewarding those who have made significant contributions in their lives already.

The Australian and New Zealand Intensive Care Society’s (ANZICS) guiding principles for complex decision making during the COVID-19 pandemic^3^ advise patient assessment by at least 2 senior intensivists considering the patient’s likelihood of surviving based on their acute illness severity, the independent prognoses of their comorbidities, and their likelihood of long-term survival. If these are equal, they advise it may be ethically justifiable to consider the following:

- supporting patients belonging to groups subjected to social deprivation and disadvantage as a means of redressing their vulnerability;
- considering that adults with caring responsibilities be prioritised;
- advocating that younger patients who have lived through fewer life stages are prioritised over older patients; and
- supporting individuals who undertake front-line patient care . . . based on the principle of reciprocity.^3^

In a 2017 study, Cheung et al.^9^ ascertained that most Australians supported active decision making in an influenza pandemic scenario (43% preferred a senior doctor make this decision and 39% favored the use of a triage protocol). Given this preference for active decision making, it is important to know whether most Australians would agree with the approach advocated in the ANZICS guidelines. When a person is likely to die because of rationing due to a lack of resource availability, this decision must be one that is consistent with community values. The aim of this experiment was to establish whether these key decision-making attributes supported by the ANZICS guidelines aligned with those of a sample of the Australian general population.

## Method

### Study Design

Members of the general population were presented with a scenario in which the COVID-19 pandemic had surged, and the adult ICU was oversubscribed. Respondents were asked to imagine that they were lay members on the hospital ethics committee responsible for recommending which of 2 patients should receive the last bed, with the patient who did not receive an ICU bed likely to die. Patients were described according to 5 attributes.

The attributes outlined in the ANZICS guidelines that could be used to discriminate between patients in an ICU bed rationing situation were considered for inclusion in the study. All of these attributes were included (some in collapsed form via *chance of surviving if they get an ICU bed)*, except for social deprivation, as we could not see how to include this without including “irrelevant and discriminatory considerations such as sex, sexual orientation, religion, disability, social status, personal connections, wealth, citizenship, insurance status, ethnicity or race.”^3^

Scenario, attributes, and levels were finalized through discussion and piloting with both medical and nonmedical respondents and refined over multiple rounds of feedback.

The attribute levels chosen for *age* ranged from 25 to 85 y in 10-y increments.

The levels for *occupation* were employed either as a frontline health care worker or employed but not a health care worker; unemployed either long term or as a result of the pandemic; home duties; or retired.

*Social role* reflected the idea that caregiving responsibilities are something that might be used to discriminate in favor of one patient over another and included caregiver for an adult dependent; parent with a youngest child less than 5 y, between 5 and 12 y, or between 13 and 18 y; or no dependents.

*Health status prior to infection* was rated as excellent, very good, good, fair or poor, in line with the Australian Bureau of Statistics standard descriptions for self-reported health status.^10^

*Chance of surviving if they get an ICU bed* (hereafter “prognosis”) was described as a score calculated by a doctor from a combination of factors that might include a person’s vital signs and other features such as blood tests or imaging investigations. Because of the rough nature of such tools, we elected to use only 3 levels for this attribute. These were 5%, 25%, and 50%. The patients were described as being likely to die if they were admitted into a general ward. Being admitted, instead, into the ICU increases the chance of survival. But for a patient so sick as to be likely to die in the general ward, being admitted into intensive care is unlikely to increase the chance of survival beyond 50%, and so we used 50% as the highest level.

After each choice, respondents were asked whether they believed it would have been fairer to allocate the bed randomly rather than to choose which patient received the bed. We hypothesized that there would be a role for random allocation as suggested in the ANZICS guidelines. Specifically, we hypothesized that patients with equal prognoses would be more likely to be randomly allocated than those with different prognoses.

**Figure 1:**
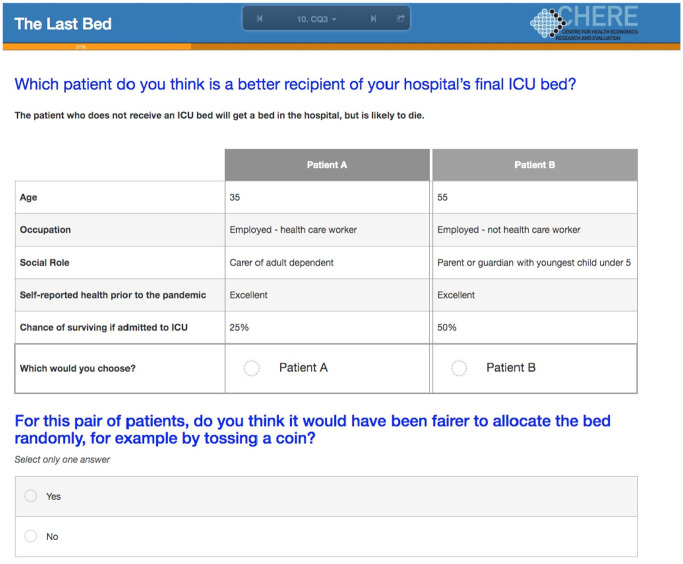
Example of one of the choice tasks as it appeared in the survey.

One of the choice tasks used in the survey appears in Figure 1.

### Designed Experiment

The experiment consisted of 98 choice tasks, constructed by taking a fractional factorial design for eleven 7-level attributes,^11^ using the first 5 attributes to define the first patient in a pair and the next 5 attributes to define the second patient in each pair. The levels were collapsed within each attribute to obtain the correct number of each, as indicated above. Because respondents can typically complete only about 15 choice tasks,^12^ the 98 tasks were subdivided into 7 versions, each of 14 choice tasks, using the 11th attribute, and respondents were randomly allocated to complete one version. Restrictions on age and occupation pairings (such as those aged 75 or 85 y having to be retired) and on age and social role pairings (such as older people not being caregivers for very young children and those aged 25 y not being caregivers for teenagers) were implemented. To avoid it being possible for respondents to always choose based on one attribute only, for instance age or prognosis, all respondents saw some choice tasks in which one or more of the attributes were presented at the same level in both options.

The actual set of 98 choice tasks used is available from the authors on request.

### Other Questions

We collected demographic information (age, gender, education, state of residence, general health, occupation, caregiving status, relationship status, and previous committee experience) and asked follow-up questions to assess respondent engagement, the difficulty respondents found in completing the discrete-choice experiment, and internal consistency. We also asked respondents their nominally preferred method of allocation.^9^

### Sample Size

Various approaches to determining the required sample size for a discrete-choice experiment have been proposed.^13^ For this study, we elected to collect at least 40 responses per choice task^14^ and a total sample of 300 respondents.

### Study Sample Selection

The survey was hosted by Survey Engine (www.surveyengine.com), and a general population sample of 300 Australians was recruited through an online survey panel provider, Toluna Australia. Age-gender quotas were used to ensure the proportions in each gender and in age group (18–29, 30–39, 40–49, 50–59, 60–69, and 70 y and older) matched the Australian adult population. Data were collected from July 3, 2020, to July 7, 2020, for the pilot study and then from July 9, 2020, to July 14, 2020, for the remaining respondents. This coincided with rising case numbers in the state of Victoria and tightening of restrictions, although it was before Victoria moved to its stage 4 lockdown.^15,16^

### Statistical Analysis

Initially, the multinomial logit model (MNL) was used to investigate the effect of the patient characteristics on changes in preferences for bed allocation. This model assumes that all respondents have the same preferences. To investigate this assumption, 2 models that allow for preference heterogeneity were also considered.

In the mixed logit model (MIXL), each respondent is assumed to have their own preference parameter, and that parameter is assumed to come from an underlying distribution of preference parameters. It is the parameters of this distribution that are estimated. As is commonly done, we assumed that the underlying distribution of the preference parameters is multivariate normal.

Latent class models assume that the underlying distribution for the parameters is discrete; that is, it is assumed that there are different decision-making strategies used by groups (often called classes) of respondents in a sample but that within each of these classes, responses are homogeneous. A model with Q = 3 classes, for instance, assumes that there are 3 different approaches to decision making in a sample and estimates the parameters for each of these classes based on the responses people have given to the choice tasks. Further details of the models can be found in the supplementary material.

Model selection was based on the Bayesian information criterion (BIC). The models were fitted in R.^17–21^

In the latent class model, individuals were assigned to a class based on their highest posterior probability of class membership; that is, predicted class membership is based on the observed choices of the respondent.^22^ The distribution of these probabilities is one measure of how well the model does in differentiating the classes.^23^ We also fitted a multinomial logit model to these assigned classes to identify respondent characteristics that predict class membership.

Demographic comparisons were made using the chi-squared test, and comparisons of answers to Likert-type items by the different classes were made using the Kruskal-Wallis test. Comparisons of the time taken to complete the survey by different classes were made using one-way analysis of variance.

## Results

### Sample

Of 882 eligible respondents who entered the survey, 440 were excluded because the quota for their age-gender category was already full. Of the remaining 442 eligible respondents, 310 completed all choice questions. Subsequently, 4 of these respondents were excluded, 2 because they had random strings of letters in open-text answers, suggesting completion by a bot, and 2 because their stated selection strategy was to choose the first option. The remaining 306 respondents are included in the descriptions and analyses below.

The demographic characteristics of the respondents are given in Table 1.

**Table 1:** Characteristics of Respondents

Characteristic	Sample (*N* = 306)	All Australians^a^
Female	153 (50%)	51%
Age, y		
18–29	63 (20.6%)	21.8%
30–39	55 (18.0%)	18.6%
40–49	52 (17.0%)	16.6%
50–59	47 (15.4%)	15.6%
60–79	45 (14.7%)	13.2%
≥70	44 (14.4%)	14.2%
Self-assessed health		
Poor	12 (3.9%)	4%
Fair	30 (9.8%)	11%
Good	113 (36.9%)	28%
Very good	108 (35.3%)	35%
Excellent	43 (14.1%)	21%
State		
NSW	107 (35.0%)	31.9%
Vic	81 (26.5%)	26.1%
Other	118 (38.6%)	42.1%
Caregiving status^b^		
Yes, full-time or nearly full-time	86 (28.1%)	
Yes, about half the time	22 (7.2%)	
Yes, less than have the time	11 (3.6%)	
No dependents	187 (61.1%)	

a Australian Bureau of Statistics (June 2019) 3101.0–Australian Demographic Statistics December 2018.

b No data available for the general population.

After the first 49 eligible respondents had completed the survey, data collection was paused, and the answers to the question about whether the number of choice tasks was acceptable were analyzed. Because more than 70% (95% confidence interval [59%, 84%]) of the pilot respondents thought that the number was about right, or fewer than they could have answered, we continued to use the same versions of 14 choice tasks for the remainder of the collection.

### Analysis of the Choice Questions

We fitted the multinomial logit model, the MIXL. and the latent class models with Q = 2, Q = 3, and Q = 4 classes.

**Figure 2:**
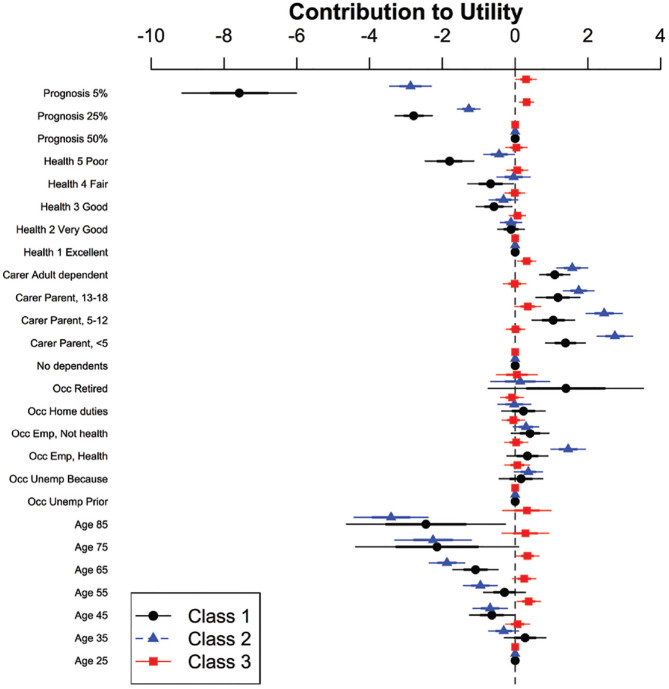
Parameter estimates from the latent class model with 3 classes. The attributes were dummy-coded, and the reference levels were age 25 y, unemployed prior to the pandemic, no dependents, health prior excellent, and prognosis 50%, each with assumed contributions to the utility of 0. Estimates for class 1 are in black, class 2 in blue, and class 3 in red.

The MIXL indicated that there was significant unobserved heterogeneity (see Table 2) in the respondent sample. We used a latent class model to examine this preference heterogeneity and identify classes with different preference structures. Based on the BIC, the best model was the latent class model with Q = 3 classes (BIC of 5096.2, cf. MIXL [uncorrelated] 5344.4 and MIXL [correlated] 5603.3). Using the latent class model with Q = 3, the classes were estimated to contain 27%, 41%, and 32% of respondents, respectively. The parameters, and corresponding 95% confidence intervals, in the 3-class model are given in Figure 2. When fitting the model, the reference levels were omitted and assumed to be 0; that is, we dummy-coded the data. A negative coefficient means that a patient with that characteristic is less likely to be chosen, all else being equal. In classes 1 and 2, there were a number of parameters that are significantly different from 0, notably prognosis, caregiving status, and older ages. In class, 3 few parameters were significant.

**Table 2:** Parameter Estimates for the MNL and MIXL Models^a^

	MNL		MIXL			
Patient Characteristic	β	SE	β	SE	SD	SE
Age 35 y	0.073	0.089	0.059	0.133	0.444	0.381
Age 45 y	−0.061	0.092	−0.154	0.13	0.119	0.606
Age 55 y	−0.159	0.091	−0.302*	0.138	0.364	0.434
Age 65 y	−0.401***	0.092	−0.696***	0.141	0.657**	0.247
Age 75 y	−0.699***	0.198	−1.192***	0.3	0.337	0.491
Age 85 y	−0.889***	0.194	−1.702***	0.313	1.061***	0.28
Unemployed due to	−0.086	0.094	0.029	0.139	0.577	0.378
Employed, HCW	0.133	0.086	0.396**	0.131	0.546	0.291
Employed, Not HCW	−0.025	0.084	0.066	0.121	0.021	0.581
Home duties	−0.067	0.09	0.066	0.141	0.002	0.669
Retired	0.198	0.169	0.398	0.257	1.563***	0.173
Parent of child <5 y	0.680***	0.071	1.198***	0.131	1.009***	0.183
Parent of child aged 5–12 y	0.963***	0.09	1.511***	0.176	1.235***	0.302
Parent of child aged 13–18 y	0.534***	0.086	0.892***	0.145	0.723*	0.333
Carer, adult dependent	0.543***	0.07	0.863***	0.115	0.674**	0.259
Health very good	0.103	0.062	0.08	0.087	0.252	0.233
Health good	0.011	0.077	−0.056	0.115	0.023	0.514
Health fair	0.100	0.082	0.113	0.124	0.645*	0.279
Health poor	−0.062	0.081	−0.318*	0.129	1.134***	0.238
Prognosis 25%	−0.524***	0.052	−0.896***	0.095	0.988***	0.15
Prognosis 5%	−1.18***	0.081	−2.264***	0.2	2.585***	0.28

a MNL, multinomial logit model; MIXL, mixed logit model; Unemployed due to – unemployed due to the pandemic; Employed, HCW – employed as a frontline healthcare worker; Employed, Not HCW – employed but not as a frontline healthcare worker. Health status is prior to contracting COVID-19. Prognosis is the probability of surviving to hospital discharge if given a bed in the intensive care unit.

*** Significant at *P* = 0.001.

** Significant at *P* = 0.01.

* Significant at *P* = 0.05.

For each respondent, we calculated the posterior probability of class membership for each of the 3 classes and assigned respondents to the class for which they had the highest posterior probability.^22^ These highest posterior probabilities of class membership were generally high, with medians of 0.96 (class 1), 0.98 (class 2), and 0.99 (class 3), and all were greater than 0.5.

**Figure 3:**
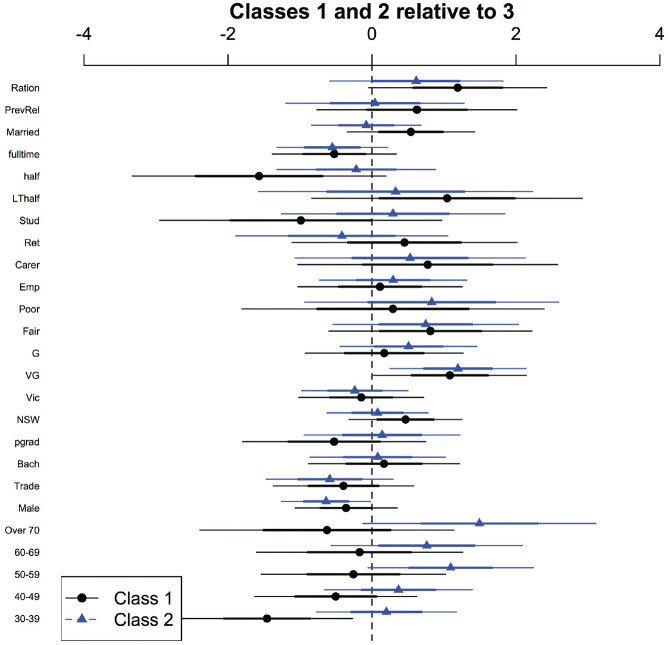
Demographic characteristics and class membership: relative risk ratios with class 3 as base. Relative risks ratios, relative to class 3, are in black for class 1 and in blue for class 2. The base levels are 18 to 29 y for age, female for gender, high school or less for education, states other than NSW and Victoria for state of residence, excellent for current health, unemployed for employment status, no carer responsibilities for caregiving status, currently unpartnered for marital status, and no rationing experience for rationing.

The MNL model to investigate which of the individual characteristics (age, gender, highest level of education, state of residence, self-reported health, occupation, caregiving status, relationship status, and prior experience of rationing scarce resources^i^) might predict class membership is presented in Figure 3 as relative risk ratios and 95% confidence intervals. Relative to class 3 (the class with an apparently random choice strategy), class 1 and 2 members are more likely to assess their health status as very good, class 2 members are marginally less likely to be male, and class 1 members are less likely to be aged 30 to 39 y (and thus more likely to be in the base age group of 18–29 y). However, overall, there was little significant difference between the individual characteristics of the classes, at least based on the demographic information that we collected.

### Analysis of Follow-up Questions

After each choice task, we asked respondents whether it would have been fairer to allocate the bed randomly, for example, by tossing a coin. Given the answers to these questions, Table 3a reports the 2 patient pairs with the lowest rate of random allocation. and Table 3b reports the 2 patient pairs with the highest rate of random allocation. Rates of random allocation were no different when prognosis was equal as compared with when it differed, but rates of random allocation were significantly greater in class 3 than in classes 1 or 2 (Table 4, more details in the supplementary material).

**Table 3a:** Choice Sets with the Lowest Rate of Random Allocation, with Responses by Class

	Age	Occupation	Caregiver Status	Health Prior	Prognosis	Class 1	Class 2	Class 3
1	75	Retired	Parent of child aged 13–18 y	Good	25%	13 (100%)	17 (65%)	2 (29%)
	35	Unemployed due to pandemic	No dependents	Excellent	5%	0 (0%)	9 (35%)	5 (71%)
Preferred random allocation						0%	19%	43%
2	55	Home duties	Parent of child aged <5 y	Good	50%	10 (91%)	17 (100%)	5 (33%)
	75	Retired	Parent of child aged 13–18 y	Good	25%	1 (9%)	0 (0%)	10 (67%)
Preferred random allocation						0%	12%	40%

**Table 3b:** Choice sets with the Highest Rate of Random Allocation, with Responses by Class

	Age	Occupation	Caregiver Status	Health Prior	Prognosis	Class 1	Class 2	Class 3
3	25	Health care worker	Carer of adult dependent	Very good	25%	2 (18%)	13 (76%)	10 (67%)
	65	Unemployed prior	Parent of child aged <5 y	Good	50%	9 (82%)	4 (24%)	5 (33%)
Preferred random allocation						27%	35%	87%
4	25	Health care worker	Parent of child aged <5 y	Very good	50%	8 (89%)	16 (94%)	8 (44%)
	75	Retired	Carer of adult dependent	Excellent	50%	1 (11%)	1 (6%)	10 (56%)
Preferred random allocation						33%	35%	78%

**Table 4:** Rates of Random Allocation by Class When Prognosis Is the Same or Different

	Random Allocation When Prognosis Equal	Random Allocation When Prognosis Different
Class 1	100/450 (22.2%)	152/726 (20.9%)
Class 2	192/679 (28.3%)	316/1099 (28.8%)
Class 3	292/530 (55.1%)	468/800 (58.5%)

After completing all choice tasks, we asked respondents to indicate the attribute that had been most important to them when making their choices and the one that had been least important. The results are summarized in Table 5. Prognosis was stated to be the most important attribute and occupation the least important, and these are consistent with the parameter estimates from the choice model for those in classes 1 and 2. There was no significant difference between the classes in the time taken to complete the survey, in the answer to questions about how they decided which patient to choose, how difficult it was to tell the patients apart, and how difficult it was to choose. Details are available in the supplementary material.

**Table 5:** Number of Respondents Who Chose a Given Attribute Pair as Being the Most Important and the Least Important in Patient Selection

	Least Important					
Most important	Age	Occupation	Social Role	Health	Prognosis	Row Totals
**Age**	0	40	24	10	5	79
**Occupation**	4	0	5	5	0	14
**Social role**	4	23	0	10	3	40
**Health**	3	7	11	0	3	24
**Prognosis**	15	72	45	17	0	149
Column totals	26	142	85	42	11	306

When it came to choosing who should decide, the number of respondents choosing each of the possible answers is given in Table 6. (This table is based on the 257 respondents in the second collection only. In the initial collection, we inadvertently omitted the final possible answer.) These values are significantly different (*P* < 0.001) from answers to the same question reported in Cheung et al.^9^ This may be because the respondents to our survey answered the question after completing 14 tasks in which they had been asked to choose between patients or because the survey was answered during a pandemic. Active choice strategies (letting a senior doctor decide or using a set of criteria) are significantly different between the classes (*P* = 0.009), with class 1 having significantly more respondents in support of active decision making than expected and class 3 having significantly fewer.

**Table 6:** Number of Respondents Who Chose Each of the Preferred Methods for Bed Prioritization, in Answer to a Multiple-Choice Question^a^

Preferred Prioritization Strategy	Whole Sample	Class 1	Class 2	Class 3
Use a first-come, first-served approach to decide	30	6	10	14
Let a senior doctor decide	67	21	28	18
Use a set of criteria or rules that have been developed by the health department to decide	99	34	42	23
Use random selection to decide	6	0	3	3
Use a patient’s ability to pay to decide	5	1	1	3
Use the importance of the patient to decide	11	4	4	3
I’m not sure which method I think is the best	39	6	19	14

a Only 1 response was allowed per respondent.

### Free-Text Reponses

Of about 80 ideas reflected in general comments at the end of the survey, nearly two-thirds felt that the study was interesting, challenging, or thought provoking. One quarter commented on how difficult the decision making is, especially when imagining the scenario as real patients or on the challenge of having to “play God.” One in 10 respondents invoked the hope that it never comes to this in Australia.

## Discussion

In this discrete-choice experiment using an age-gender representative random sample of the Australian general population, a number of themes emerged about patient prioritization for ICU bed space in the event of a catastrophic surge in COVID-19 caseload, for example, in the wake of a highly transmissible mutant strain finding its way into the community. Importantly, the sample suggests that Australians are heterogeneous in their decision making about ICU bed utilization, with 3 classes in the latent class model that provided the best fit for describing the data.

These classes are best considered as 2 classes with interpretable decision-making strategies and a third class with no apparent bed-prioritization strategy.

From Figure 2, we can see that for respondents in class 1, patients with a worse prognosis are much less likely to be prioritized for a bed. Age 65 y or older and a prior health state of good, fair, or poor also decreased the probability of being prioritized for a bed (age 75 y significant only at 0.1 level). Any caregiving responsibility slightly increased the probability of being allocated the final bed.

Respondents in class 2 similarly preferred patients with a better prognosis but less strongly than class 1. This class more strongly discriminated in favor of younger patients, with an age of 45 y or greater being associated with a decreased probability of being prioritized for a bed, with this parameter becoming more negative with increasing age. Like class 1, this class discriminated in favor of anyone with a caregiving responsibility, but unlike class 1, health state prior was not significant unless the patient’s health state had been poor, in which case it counted against them. This class of respondents discriminated in favor of patients employed as frontline health care workers.

The third class had no large estimates for any parameters, and some parameters were inexplicable, such as a slight preference for people with a 5% probability of surviving to hospital discharge as compared with those with a better prognosis. It is possible, despite the description at the start of the survey that explained prognosis, that some respondents did not understand that a higher value meant a greater chance of survival. Another feature of this third class was a significant preference for more random patient-prioritization models of care. In this class, only 52.6% responded that their preferred decision-making strategy was for a senior doctor to decide or to use a set of criteria to triage patients. The remaining respondents preferred prioritization based on order of arrival, random bed allocation, patient importance, patient capacity to pay, or were unsure. This is in contrast to 76.4% of respondents in class 1 and 65.4% in class 2 (Table 6). Class 3 respondents were also more likely to answer that it would have been fairer to allocate the bed randomly after each choice task (Table 2, also supplementary appendix).

As we see in Tables 3a and 3b, choice set 3 demonstrates the different attribute prioritization in practice for classes 1 and 2, illustrating the general preference in favor of patients with a higher prognosis for class 1 as compared with the general preference for younger patients in class 2 (noting that occupation may also drive some of this decision for class 2).

The ANZICS guidelines suggest that an active decision-making strategy be used. This strategy may reasonably be implemented as 2 senior intensivists making bed-rationing decisions informed by these guidelines or facilitated by a more distanced triage committee with assessment and advice from treating intensivists. These guidelines order the probability of survival to hospital discharge and underlying comorbidities as variables that should be used in the first instance to discriminate between patients when ICU resources are overwhelmed, with the other variables that we studied to be considered only if the former did not allow discrimination between the patients. This is a similar decision-making strategy to respondents who fell into class 1 and broadly similar to those in class 2, although this class tends to more strongly favor younger patients. The guidelines probably underplay the value placed on caregiving responsibilities by respondents, and decision makers would be in line with community values if caregiving status is used to discriminate between patients should rationing be necessary.

Limitations of this study include that the probability of survival to hospital discharge is not something that can really be so clearly delineated. Predictions of survival are always inexact, and the score respondents were asked to imagine was a hypothetical score representing the sort of score that clinicians may use or otherwise estimate from their gestalt. Whether or not decision makers could rely on such an estimate to choose between patients may be harder in reality, because of the lack of certainty.

Other limitations of this study include the abstract nature of decision-making tasks on a screen, which may result in different decision patterns than would truly be observed in lay members of a triage committee. It seems likely that this may explain some of the decision making seen in class 3 respondents, who may have found it difficult to engage with the choice tasks.

Future research could investigate whether a sample of health care workers, especially intensivists, yielded similar results. One might hope that health care workers might be more consistent in their decision making and be more likely to fall into classes 1 or 2 or to subscribe uniformly to a random method of allocation.

## Conclusion

For about two-thirds of the population, a higher probability of surviving to hospital discharge, a younger age, a better health state prior to contracting COVID-19, and being a caregiver increase the likelihood of being preferentially allocated the final ICU bed. About 40% of this sample also gives preference to frontline health care workers. About one-third of respondents use a relatively random decision-making strategy.

Overall, this study found that the ANZIC’s guiding principles are relatively consistent with community values but that a greater focus could be placed on discriminating in favor of younger patients and those with caregiving roles, when the probability of survival is relatively similar. Let us hope, however, that we never face this situation and that our vaccination rollout is swift and effective.

## Supplemental Material

sj-pdf-1-mdm-10.1177_0272989X21996615 – Supplemental material for Who Gets the Last Bed? A Discrete-Choice Experiment Examining General Population Preferences for Intensive Care Bed Prioritization in a Pandemic Click here for additional data file. Supplemental material, sj-pdf-1-mdm-10.1177_0272989X21996615 for Who Gets the Last Bed? A Discrete-Choice Experiment Examining General Population Preferences for Intensive Care Bed Prioritization in a Pandemic by Amelia E. Street, Deborah J. Street and Gordon M. Flynn in Medical Decision Making

## Notes

i. We asked respondents, “Have you ever been in a situation or part of a committee that has had to decide how to allocate limited resources fairly? Examples might include being involved in a committee at your local school, in a local sporting club or in some other community group, or you might have had to help allocate resources as part of your job.”
