# Supplementary material for: Who Gets the Last Bed? A Discrete-Choice Experiment Examining General Population Preferences for Intensive Care Bed Prioritization in a Pandemic
Source: Med Decis Making. 2021 Mar 4:0272989X21996615. doi: 10.1177/0272989X21996615 (PMC7941140; doi:10.1177/0272989X21996615)
Supplement: sj-pdf-1-mdm-10.1177_0272989X21996615 – Supplemental material for Who Gets the Last Bed? A Discrete-Choice Experiment Examining General Population Preferences for Intensive Care Bed Prioritization in a Pandemic [file sj-pdf-1-mdm-10.1177_0272989X21996615.pdf]

Supplementary Material

Who Gets The Last Bed?

Amelia E. Street, Deborah J. Street and Gordon M. Flynn.

The statistical analysis

Choice models assume that individuals make choices that maximise the utility to them of the choice made.

Initially we fitted a model in which we assumed that the contribution to the utility of a given level of a given attribute was the same for all respondents; that is, we assumed homogeneity across individuals. Thus the utility derived by individual α from choosing alternative i in choice sets c is given by

$$U_{i\alpha c} = \beta x_{i\alpha c} + \epsilon_{i\alpha c}, \;\; i = 1, 2; \; \alpha = 1, \ldots, N; \; c = 1, \ldots, 98,$$

where $x_{i\alpha c}$ is the vector of attribute levels for option i in choice set c, β is the common vector of utility weights and $\epsilon_{i\alpha c}$ is the idiosyncratic error which we assumed to be distributed iid extreme value. With this notation, the probability that option 1 is chosen is

$$P(\text{option 1 is chosen}) = P(U_{1\alpha c} > U_{2\alpha c}).$$

With our assumptions it can be shown (eg, Train (2009)) that

$$P(\text{option 1 is chosen}) = \frac{\exp(\beta x_{1\alpha c})}{(\exp(\beta x_{1\alpha c}) + \exp(\beta x_{i\alpha c}))}.$$

We considered two extensions of the multinomial logit model to allow for preference heterogeneity - the mixed logit (MIXL) model and a latent class model.

In the MIXL model the utility is given by

$$U_{i\alpha c} = (\beta + \eta_\alpha) x_{i\alpha c} + \epsilon_{i\alpha c}, \;\; i = 1, 2; \; \alpha = 1, \ldots, N; \; c = 1, \ldots, 98,$$

where β is the population mean attribute utility weights and η_α is the vector of individual specific deviations from the mean. We assumed that the $\eta_\alpha$were multivariate normal with mean 0 and with covariance matrix Σ. We fitted two MIXL models - one in which we assumed that the entries in η_α were independent (and so all off-diagonal entries in Σ were 0) and one in which this assumption was relaxed.

We used the latent class model to investigate a discrete distribution for preference heterogeneity. In this model each respondent is assumed to belong to one of Q latent classes, where preferences differ between classes but are assumed to be homogeneous within classes. The possible values for β are β_q, $q = 1, \ldots, Q$, and $\beta = \beta_q$ with probability ω_q where $\Sigma_q \omega_q = 1$ and $\omega_q > 0 \; \forall q$.

For the model with $Q = 3$ classes, for instance, looking at Figure 2, we see that people in both classes 1 and 2 feel that, all else being equal, someone with a prognosis of

5% has a lower utility than someone with a prognosis of 50%. But in class 1 the probability of that person being chosen is $e^{-7.58}/(1+e^{-7.58}) = 0.0005$ whereas in class 2 it is $e^{-2.88}/(1+e^{-2.88}) = 0.05$.

The Bayesian Information Criterion for each of the models we fitted is given in the table below.

Model	BIC
MNL	5433.6
MIXL (uncor)	5344.4
MIXL (corr)	5603.3
LC, $Q = 2$	5131.8
LC, $Q = 3$	5096.2
LC, $Q = 4$	5229.4

Analysis of the follow-up questions

When asked how they decide which patient was the better recipient, 130 respondents said that they considered all features of each patient, 121 said that they considered those features which differed, 42 only considered features which were most important to them and 13 used some other strategy. There was no significant difference between the three classes.

The majority of respondents (230/306) agreed or strongly agreed that it was easy to distinguish between the patients while 28 disagreed or strongly disagreed that it was. There was no significant difference between the three classes.

A small majority of respondents (161/306) agreed or strongly agreed that they could easily choose between the patients, while 81 disagreed or strongly disagreed that they could. The observed chi-squared value is 52.6 on 2 degrees of freedom and so significantly more people found it easy to choose than did not. There was no significant difference between the three classes.

Random allocation of beds

The randomisation rate was significantly different by class. To establish this, we constructed a three-way contingency table with factors class (with three levels), prognosis (with two levels, equal and not equal) and random allocation (with two levels, yes and no). We fitted a model in which class, prognosis and random allocation are mutually independent and one in which class and randomisation were dependent given the prognosis. This was a significant improvement on the model of mutual independence (p value less than 0.000001; AIC 107.72 cf AIC 510.08).

Additional References

Train, K. E. (2009). *Discrete Choice Methods with Simulation.* Cambridge University Press.
